# Adherence to denosumab therapy and fracture risk associated with drug withdrawal: a retrospective study

**DOI:** 10.3325/cmj.2025.66.334

**Published:** 2025-10

**Authors:** Hande Özdemir, Nur Kakilli, Filiz Tuna, Buket Yılmaz Bülbül, Mehmet Çelik, Selçuk Korkmaz, Derya Demirbağ Kabayel

**Affiliations:** 1 Department of Physical Medicine and Rehabilitation, Faculty of Medicine, Trakya University, Edirne, Turkey; 2 Department of Endocrinology and Metabolic Diseases, Faculty of Medicine, Trakya University, Edirne, Turkey; 3 Department of Biostatistics and Medical Informatics, Faculty of Medicine, Trakya University, Edirne, Turkey; Özdemir et al: Adherence to denosumab therapy and fracture risk associated with drug withdrawal

## Abstract

**Aim:**

To assess the prevalence of fragility fractures after denosumab discontinuation and to identify the factors affecting treatment adherence.

**Methods:**

We retrospectively reviewed the medical records of 3876 osteoporosis patients who were treated with denosumab at Trakya University Osteoporosis Clinic between 2015 and 2021. A total of 210 patients who received at least two regular doses of denosumab were eligible for inclusion. Patients were categorized as denosumab-adherent and denosumab-non-adherent. Adherence was defined as receiving the six-month scheduled dose with a maximum delay of up to eight weeks.

**Results:**

Overall, 124 (59.05%) patients were denosumab-adherent and 86 patients (40.95%) were denosumab-non-adherent. New fragility fractures were identified in 32 patients: 17 in the denosumab-adherent group and 15 in the denosumab-non-adherent group. The groups did not significantly differ in the risk or types of new fractures, irrespective of patients’ fracture history.

**Conclusions:**

Our findings suggest that some fractures after denosumab discontinuation may stem from the natural progression of osteoporosis rather than from the rebound effect. Still, unscheduled treatment discontinuation should be prevented.

Denosumab is a novel anti-resorptive agent associated with a low incidence of fractures and a low rate of adverse events. It produces a continued increase in bone mineral density (BMD) that does not plateau for up to 10 years (1). However, its potent anti-resorptive effects reverse rapidly upon treatment cessation, a phenomenon referred to as the “rebound effect” (2,3).

Besides the rebound effect following denosumab discontinuation, fragility fractures may also arise as a result of the natural progression of osteoporosis, particularly in patients at high risk for fractures. Research on this topic remains limited, and the reports show conflicting results regarding the timing, number, and location of rebound fractures. This study aimed to assess the prevalence of fragility fractures in patients who discontinued denosumab therapy for any reason and to identify the factors influencing treatment adherence.

## PATIENTS AND METHODS

### Study design and patient population

This retrospective study was conducted at the Department of Physical Medicine and Rehabilitation and the Department of Endocrinology of Trakya University between December 2022 and April 2023. The study protocol was approved by the Science Research Ethics Committee of Trakya University Faculty of Medicine. Patient consent was waived due to the retrospective design of the study. The study was registered at ClinicalTrials.gov (ID: NCT06414616) and adhered to the principles of the World Medical Association Declaration of Helsinki.

The electronic medical records of patients who started denosumab (Prolia®) therapy between 2015 and 2021 were retrospectively evaluated. The inclusion criteria were as follows: 1) having received at least two consecutive doses (60 mg subcutaneously) of denosumab, with an interval of fewer than 8 months between the doses (as it is a minimum dosage to observe the anti-fracture effects of the medication) (4); 2) having had at least one radiological image of the lumbar and thoracic spine taken before the initiation of denosumab 3) having BMD and T-score assessed using dual-energy x-ray absorptiometry, and having fracture risk assessment tool scores calculated before the initiation of denosumab. Exclusion criteria were 1) fractures due to cancer, trauma, or Paget’s disease; 2) monthly denosumab injections to prevent bone metastasis; 3) age under 18 years, pregnancy, and premenopause.

We recorded data on age, sex, age at menopause, parity, marital status, occupation, height, weight, body mass index (BMI), family history of fragility fractures, the presence of baseline fragility fractures, comorbidities, use of steroids, smoking status, the presence of secondary osteoporosis, tea/coffee/alcohol consumption, the intake of dairy products, any previous osteoporosis therapy, the treatment agent used before denosumab, the number of denosumab injections received, the duration of denosumab withdrawal, and the reason for denosumab discontinuation.

Radiological images were examined to determine whether any fractures were present before, during, or after denosumab treatment. The number and location of the fractures were recorded. The number of denosumab doses administered was determined by reviewing the hospital’s electronic prescription and medical records.

Patients who missed the injections for more than two months were classified as denosumab-non-adherent, while those who received their injections regularly were classified as denosumab-adherent. An increased risk of vertebral fractures (VFx) has been reported as early as 4-8 weeks after a missed injection (5,6). In the current study, adherence was defined as receiving a scheduled six-month dose with a maximum delay of up to eight weeks, consistent with previous studies (7,8). This definition is based on the observation that multiple VFx are often reported 2-10 months after a delay in the dosing schedule (9).

The duration of the off-treatment period after denosumab discontinuation was calculated as the interval between six months after the last injection and the patient’s admission date. Patients in the denosumab-non-adherent group did not receive any alternative osteoporosis treatment during the off-treatment period. We also determined the differences in clinical features between patients with and without a history of fractures before starting denosumab.

### Study outcomes

The primary outcome was the prevalence of new fragility fractures, including single or multiple VFx and non-vertebral fractures (NVFx) (any fragility fractures such as hip fractures), as defined radiologically. Hip fractures were classified as those occurring in the femoral neck, intertrochanteric region, or subtrochanteric region (4). Vertebral compression fractures were defined according to the Genant classification as at least a 20% reduction in the height of any of the anterior, middle, or posterior aspects of a vertebra compared with the height of the nearest normal vertebra. Multiple VFx were defined as two or more VFx (10). The secondary outcome was the exploration of factors that may influence denosumab adherence or non-adherence.

### Statistical analysis

Continuous variables were summarized as means and standard deviations (SD), and categorical variables as frequencies and percentages. The normality of distribution was tested with a Shapiro-Wilk test. Differences between the groups in continuous variables were assessed with a *t* test, while differences in categorical variables were assessed with a Pearson χ^2^ test. Two-tailed *P* values lower than 0.05 were considered significant. Statistical analysis was performed with SPSS Statistics 23.0 (IBM Corp., Armonk, NY, USA).

## RESULTS

### Participant characteristics

The medical records of 3876 patients who started denosumab therapy between 2015 and 2021 were reviewed. A total of 210 patients were eligible for inclusion: 207 postmenopausal women (98.6%) and 3 men (1.4%). The mean age was 69.44 ± 9.35 years. The most commonly observed comorbidities were hypertension (62.4%) and malignancy (29.5%). Baseline fragility fractures before the initiation of denosumab therapy were present in 89/210 patients (42.4%). A total of 135/210 patients (64.3%) had a history of osteoporosis medication use, with 107/210 (51%) having a history of oral bisphosphonate use. Participants received a median of 4 denosumab injections (range 2-13).

### Adherence to denosumab therapy

Out of 210 patients, 124 (59.05%) were adherent and 86 (40.95%) were non-adherent to denosumab. Most of the baseline demographic and clinical characteristics were similar between the groups (Table 1), but the denosumab-non-adherent group had significantly higher parity (*P* = 0.016) and widowhood rates (*P* = 0.010). The denosumab-adherent group had significantly more administered denosumab doses (*P* < 0.001). None of the patients reported alcohol consumption.

**Table 1:** Demographic and clinical characteristics in denosumab (DMAB)-adherent and DMAB-non-adherent patients

	Total (n = 210)	DMAB-adherent (n = 124)	DMAB-non-adherent (n = 86)	*P* value
Age (years), mean ± standard deviation (SD)	69.44 ± 9.35	68.61 ± 9.49	70.63 ± 9.07	0.125
Sex, n (%)				1.000
female	207 (98.6)	122 (98.4)	85 (98.8)	
male	3 (1.4)	2 (1.6)	1 (1.2)	
Age at menopause (years), mean ± SD	45.81 ± 5.49	45.93 ± 5.61	45.64 ± 5.32	0.700
Parity, median (min-max)	2 (0-9)	2 (0-6)	2 (0-9)	0.016
Marital status, n (%)				0.010
married	149 (71)	95 (76.6)	64 (62.8)	
single	14 (6.7)	10 (8.1)	4 (4.7)	
widow	47 (22.4)	19 (15.3)	28 (32.6)	
Occupation, n (%)				0.672
housewife	153 (72.9)	88 (71)	65 (75.6	
retired	53 (25.2)	33 (26.6)	20 (23.3)	
active working	4 (1.9)	3 (2.4)	1 (1.2)	
Height, mean ± SD	155.77 ± 7.44	155.63 ± 7.37	155.97 ± 7.57	0.748
Weight, mean ± SD	67.05 ± 11.99	66.10 ± 11.78	68.41 ± 12.23	0.170
BMI, mean ± SD	27.90 ± 5.06	27.58 ± 4.97	28.51 ± 5.20	0.287
Family history of fragility fracture, n (%)				0.693
yes	46 (21.9)	26 (21)	20 (23.3)	
no	164 (78.1)	98 (79)	66 (76.7)	
Fracture before DMAB, n (%)				0.328
yes	89 (42.4)	56 (45.2)	33 (38.4)	
no	121 (57.6)	68 (54.8)	53 (61.6)	
Comorbidities, n (%)				
diabetes	48 (22.9)	29 (23.4)	19 (22.1)	0.826
hypertension	131 (62.4)	76 (61.3)	55 (64)	0.695
dyslipidemia	26 (12.4)	16 (12.9)	10 (11.6)	0.789
hyperthyroidism	4 (1.9)	3 (2.4)	1 (1.2)	0.648
hypothyroidism	56 (26.7)	35 (28.2)	21 (24.4)	0.540
malignity	62 (29.5)	34 (27.4)	28 (32.6)	0.422
Use of steroids, n (%)				0.357
yes	22 (10.5)	15 (12.1)	7 (8.1)	
no	188 (89.5)	109 (87.9)	79 (91.9)	
Smoking, n (%)				0.533
yes	15 (7.1)	10 (8.1)	5 (5.8)	
no	195 (92.9)	114 (91.9)	81 (94.2)	
Secondary osteoporosis, n (%)				0.210
yes	111 (52.9)	70 (56.5)	41 (47.7)	
no	99 (47.1)	54 (43.5)	45 (52.3)	
Tea consumption/d (cup), median (min-max)	2 (0-20)	2 (0-20)	2 (0-10)	0.501
Coffee consumption/d (cup), median (min-max)	1 (0-5)	1 (0-5)	1 (0-3)	0.525
Consumption of dairy products, n (%)				0.144
regular	148 (70.5)	81 (65.3)	67 (77.9)	
occasional	59 (28.1)	41 (33.1)	18 (20.9)	
never	3 (1.4)	2 (1.6)	1 (1.2)	

The most common reason for denosumab discontinuation (61/86 non-adherent patients; 70.9%) was the COVID-19 pandemic. No adverse events were reported. Twenty-two out of the 86 patients (25.6%) reported other reasons for discontinuation, such as forgetting the appointment (n = 4) or preferring not to attend (n = 18) (Table 2).

**Table 2:** Treatment variables in denosumab (DMAB)-adherent and DMAB-non-adherent patients

	Total (n = 210)	DMAB-adherent (n = 124)	DMAB-non-adherent (n = 86)	*P* value
Any therapy prior to DMAB, n (%)				0.209
yes	135 (64.3)	84 (67.7)	51 (59.3)	
no	75 (35.7)	40 (32.3)	35 (40.7)	
Treatment agent prior to DMAB, n (%)				
oral bisphosphonate.	107 (51)	68 (54.8)	39 (45.3)	0.684
IV bisphosphonate.	34 (16.2)	24 (19.4)	10 (11.6)	0.097
strontium	22 (10.5)	14 (11.3)	8 (9.3)	0.742
teriparatide	5 (2.4)	2 (1.6)	3 (3.5)	0.381
Number of DMAB injections (median, min-max)	4 (2-13)	6 (2-13)	4 (2-10)	<0.001
Duration of DMAB discontinuation, (months; median, min-max)	-	-	23 (2-70)	-
Reason for DMAB discontinuation, n (%)	-	-		-
COVID pandemic			61 (70.9)	
comorbidities			3 (3.5)	
side effects			0 (0)	
other			22 (25.6)	

### Vertebral and non-vertebral fractures

New fragility fractures were detected in 32 patients. Among these, 17 patients in the denosumab-adherent group developed new fractures while on denosumab treatment, and 15 patients in the denosumab-non-adherent group developed new fractures during the off-treatment period (22 VFx in 11 patients and 4 NVFx in 4 patients).

**Figure 1:**
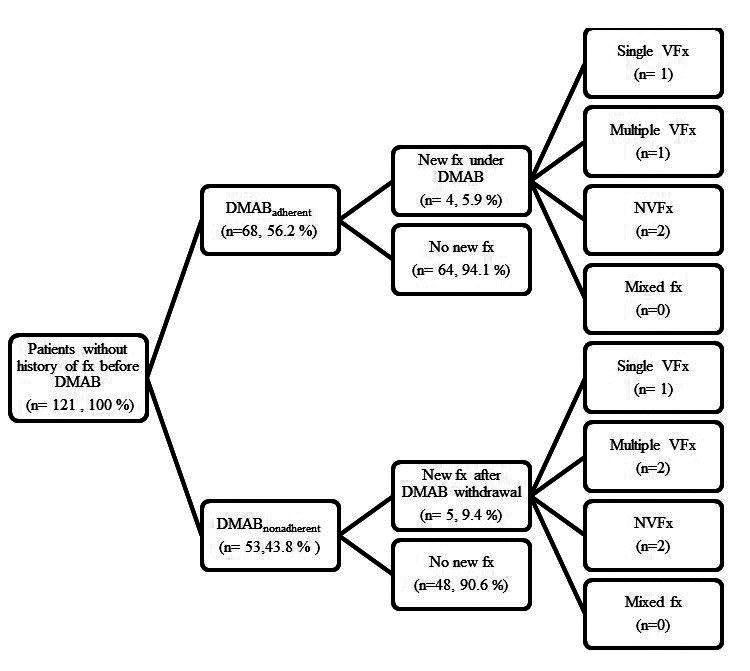
Compliance rates and fracture prevalence in patients without a history of fracture before the initiation of denosumab (DMAB). Fx – fracture, VFx – vertebral fracture, NVFx – non-vertebral fracture, mixed fx – a combination of VFx and NVFx.

In patients without a history of fractures before initiating denosumab (n = 121), the prevalence of new fragility fractures was 5.9% (n = 4) in the denosumab-adherent group and 9.4% (n = 5) in the denosumab-non-adherent group (Figure 1). In this subset of patients, the denosumab-adherent and denosumab-non-adherent groups did not significantly differ in the risk of new fracture development (risk ratio [RR] = 0.624; 95% confidence interval [CI] 0.176-2.209, *P* = 0.464). They also did not significantly differ in the risk of single VFx (RR = 1.327, 95% CI 0.085-20.687, *P* = 0.840), multiple VFx (RR = 2.600, 95% CI 0.243-27.869, *P* = 0.4298), or NVFx (RR = 1.320, 95% CI 0.193-9.051, *P* = 0.777).

**Figure 2:**
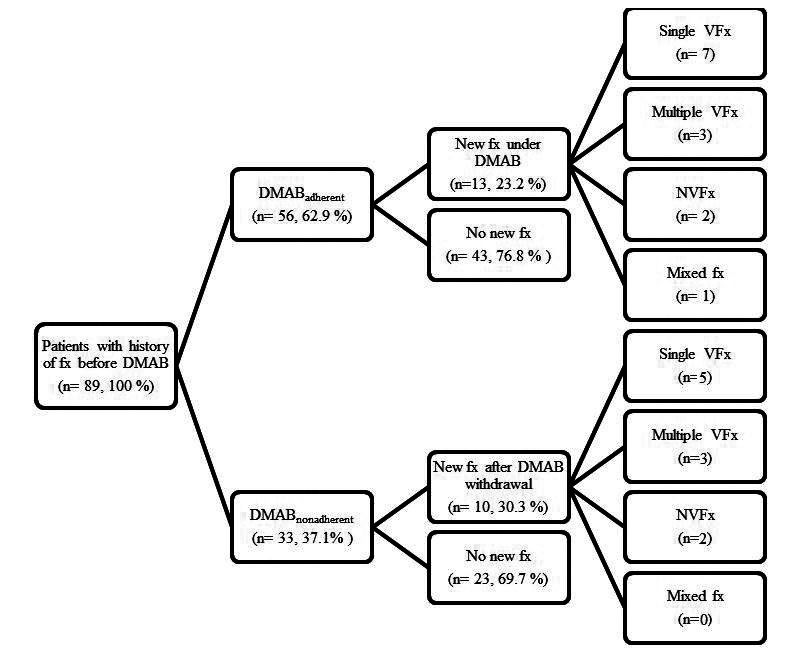
Compliance rates and fracture prevalence in patients with a history of fracture before the initiation of denosumab (DMAB). Fx – fracture, VFx – vertebral fracture, NVFx – non-vertebral fracture, mixed fx – a combination of VFx and NVFx.

In patients with a history of fractures before initiating denosumab (n = 89), the prevalence of new fragility fractures was 23.2% (n = 13) in the denosumab-adherent group and 30.3% (n = 10) in the denosumab-non-adherent group (Figure 2). In this subset of patients, the denosumab-adherent and denosumab-non-adherent groups did not significantly differ in the risk of new fracture development (RR = 0.766, 95% CI 0.379-1.548, *P* = 0.458). They also did not significantly differ in the risk of single VFx (RR = 1.276, 95% CI 0.446-3.646, *P* = 0.650), multiple VFx (RR = 1.769, 95% CI 0.385-8.141, *P* = 0.464), NVFx (RR = 1.800, 95% CI 0.270-12.011, *P* = 0.544), or mixed fractures (RR = 0.625, 95% CI 0.026-14.763, *P* = 0.770). No significant difference was observed in the prevalence of new fragility fractures in patients without a fracture history (RR = 0.624) and those with a previous fracture history (RR = 0.766; *P* = 0.780).

### The characteristics of patients who sustained fractures after denosumab withdrawal

Five patients without a history of fractures before denosumab treatment developed new fractures after discontinuation. All were postmenopausal women. The most common fracture site was the vertebra, with multiple VFx observed in 2 patients. No cases of mixed fractures (both VFx and NVFx) were reported. Among the 5 patients, 4 had a history of malignancy and 1 had rheumatoid arthritis with long-term corticosteroid use. Only 1 patient had a family history of hip fracture, and 1 patient had a history of bisphosphonate use. No fractures occurred during denosumab treatment. The number of denosumab administered doses ranged from 2 to 4 (Table 3).

**Table 3:** Clinical and densitometric characteristics of patients without a history of fracture before the initiation of denosumab (DMAB) who had new fractures after DMAB discontinuation*

Patient	Age	BMI	Sex	Site of new fracture after DMAB withdrawal	Family history of hip fracture	Comorbidities	Prior therapy	Number of DMAB injections	Duration of DMAB withdrawal (months) at evaluation	Reason of DMAB withdrawal	Pre-DMAB fracture	Pre-DMAB DXA T-score	Pre- DMAB DXA BMD
1^†^	85	28.9	F	L4	-	diabetes hypertension breast cancer Alzheimer	-	2	44	pandemic	major: 18 hip: 6.2	L1-L4: −0.1 DF:-2.7	L1-L4: 1.03 DF: 0.42
2	75	36.4	F	calcaneus	+	diabetes hypertension dyslipidemia breast cancer (use of letrozole)	-	2	45	pandemic	major: 19 hip: 5.5	L1-L4: −1.6 FN: −2.34	L1-L4: 0.869 FN: 0.586
3	62	27.1	F	distal radius	-	diabetes thyroid papilary cancer (hypothyroidism)	10 y of Aledronate	3	14	pandemic	major: 3.6 hip: 0	L1-L4: −3.5 FN: 1.3	L1-L4: 0.654 FN: 1.051
4	60	24.6	F	T12, L2, L4	-	rheumatoid arthritis (use of methotrexate and prednisolone)	-	4	10	pandemic	major: 13 hip: 2.8	L1-L4: −3.7 FN: −2.0	L1-L4: 0.644 FN: 0. 630
5	82	20.4	F	T9, T12	-	hypertension CML (use of imatinib)	-	4	36	pandemic	major: 11 hip: 5.4	L1-L4: −4.5 FN: −2.6	L1-L4: 0.550 FN: 0.556

*Abbreviations: BMI – body mass index; DF – distal forearm; FN – femoral neck; DXA – dual-energy x-ray absorptiometry; BMD – bone mineral density (g/cm^2^); CML – chronic myeloid leukemia.

†Patient 1 had bilateral total hip replacement due to coxarthrosis so distal forearm measurement was given.

Ten patients with a history of fractures before initiating denosumab developed new fractures after denosumab discontinuation. All were postmenopausal women. The most common site of fracture was the vertebra, with multiple VFx observed in 3 patients. No cases of mixed fractures were reported. Among 10 patients, 2 had a history of malignancy and 1 had systemic lupus erythematosus with long-term corticosteroid use. Three patients had a family history of hip fracture. Five patients had previously used bisphosphonate, and 1 had used teriparatide. Additionally, 2 patients had developed fractures during denosumab treatment. The number of administered denosumab doses ranged from 2 to 8 (Table 4).

**Table 4:** Clinical and densitometric characteristics of patients with a history of fracture before the initiation of denosumab (DMAB) and a new fracture after DMAB withdrawal*

Patient	Age	BMI	Sex	Site of previous fracture	Any fracture under DMAB	Site of new fracture after DMAB withdrawal	Family history of hip fracture	Comorbidities	Prior therapy	Number of DMAB injections	Duration of DMAB withdrawal (months) at evaluation	Reason of DMAB withdrawal	Pre-DMAB fracture	Pre-DMAB DXA T-score	Pre-DMAB DXA BMD
1	86	21.4	F	T12, L2	T7, T8, T9, T10	T6	-	hypertension	-	6	7	pandemic	major: 44 hip: 28	L1-L4: −3.1 FN: −4.4	L1-L4: 0.707 FN: 0.364
2	86	27.3	F	metacarpal, distal radius	-	T12, L1, L4, L5	+	hypertension hyperthyroidism	-	3	15	pandemic	major: 74 hip: 69	L1-L4: −4.0 FN: −4.5	L1-L4: 0.607 FN: 0.346
3	52	30	F	L1	-	T8	+	diabetes hypertension dyslipidemia hypothyroidism	1 y zoledronate	3	16	pandemic	major: 13 hip: 0.6	L1-L4: −2.6 FN: −1.2	L1-L4: 0.75 FN: 0.71
4	83	18.2	F	T8,T9	-	T7	-	diabetes	1.5 y teriparatide	3	19	pandemic	major: 14 hip: 3.8	L1-L4: −4.1 FN: −1.8	L1-L4: 0.599 FN: 0.648
5	66	28.1	F	distal radius	-	distal radius	-	endometrium cancer	1 y aledronate	5	15	personal preference	major: 21 hip: 8.3	L1-L4: −3.6 FN: −3.1	L1-L4: 0.65 FN: 0.50
6	62	33.3	F	femoral neck, T11, T12	-	L1,L2,L3,L4,L5	-	hypertension hypothyroidism	5 y aledronate	4	22	pandemic	major: 20 hip: 7.7	L1-L4: −1.9 FN: −3.1	L1-L4: 0.983 FN: 0.613
7	70	24.1	F	metatarsal, distal radius	-	T6	+	-	-	3	23	pandemic	major: 12 hip: 0.9	L1-L4: −2.5 FN: −0.8	L1-L4: 0.768 FN: 0.759
8	71	27.6	F	T10, T11, T12, L1, L2, L3, L4, L5	-	T8, T9	-	hypertension SLE (long term steroid use) >30 packs/y of cigarettes bypass surgery	-	2	24	pandemic	major: 56 hip: 39	L1-L4: −0.9 FN: −3.6	L1-L4: 0.951 FN: 0.447
9	79	26.7	F	L1	-	T8	-	diabetes hypertension hypothyroidism atrial fibrillation heart failure	8 y aledronate	2	32	pandemic	major: 30 hip: 12	L1-L4: −3.6 FN: −3.2	L1-L4: 0.654 FN: 0.497
10	69	31.2	F	T7, L3, L4, L5	T5	ankle	-	hypertension breast cancer (use of tamoxifen) heart failure renal failure hepatitis B carrier	3 y aldronate, 1 y zoledronate	8	10	pandemic	major: 14 hip: 2.3	L1-L4: −1.3 FN: −1.9	L1-L4: 0.90 FN: 0.63

*Abbreviations: BMI – body mass index; DF – distal forearm; FN – femoral neck; DXA – dual-energy x-ray absorptiometry; BMD – bone mineral density (g/cm^2^); SLE – systemic lupus erythematosus.

## DISCUSSION

In this study, the prevalence and type of new fractures were similar between denosumab-adherent and non-adherent patients, regardless of their history of fragility fractures. Additionally, nearly 41% of osteoporosis patients discontinued denosumab for over two months due to various reasons, primarily the COVID-19 pandemic. These patients were more likely to have higher parity or be widowed.

Contrary to expectations, the history of fractures did not significantly affect the risk of new fractures following denosumab discontinuation. Similar to our findings, Miller et al (11) did not observe an increased fracture incidence among the small number of patients who discontinued denosumab in their study. In a study by Bone et al (2), the incidence of clinical fractures 24 months after denosumab discontinuation was similar to that in the placebo group. Brown et al (12) also found no excess of fractures after denosumab discontinuation compared with placebo during the off-treatment period for up to 24 months.

Although several reports indicated an increased risk of VFx in non-persistent denosumab users (5,13-16), the risk of rebound fractures after denosumab discontinuation remains unclear. Many studies have shown that the risk of VFx significantly increases following denosumab discontinuation. These findings can be explained by the hypothesis that trabecular bone, being more rapidly affected by increased bone turnover, is more vulnerable than cortical bone (9,13,17). Conversely, a recent study found that any fractures, including hip fractures and multiple VFx, occurred more frequently in patients who discontinued denosumab compared with persistent users (18). The precise contribution of rebound phenomenon vs the natural progression of osteoporosis in high fracture-risk patients remains uncertain, which suggests that the return to the pretreatment fracture risk cannot be attributed solely to the rebound phenomenon (19). The risk factors for VFx after denosumab discontinuation include a history of VFx (12,20), longer duration of denosumab treatment (9,21), greater BMD loss (20), and the use of aromatase inhibitors (9,22). The rebound effect is not induced by a single denosumab dose (12,23), but it becomes more pronounced with an increased number of doses (24). Greater BMD gain during treatment corresponds to greater BMD loss upon discontinuation (25). Conversely, other studies reported that BMD response (26) and VFx incidence (20) were not related to the duration of denosumab treatment. Everts-Graber et al (27) associated greater BMD loss after denosumab withdrawal with younger age, lower BMI, longer denosumab therapy, and a lack of prior antiresorptive treatment, while Tripto-Shkolnik et al (18) found no association between prior bisphosphonate use and a reduced fracture risk following denosumab discontinuation. In our study, the risk of developing any type of fracture was not significantly increased following denosumab discontinuation. Furthermore, patients who experienced new fractures after denosumab discontinuation frequently used glucocorticoids and had an inflammatory disease and/or malignancy.

Modi et al reported that 48.8% and 64.3% of patients discontinued denosumab at 12 and 24 months, respectively (28). In our study, the discontinuation rate was 41%. However, since the primary reason for non-adherence was the COVID-19 pandemic, this rate may not represent non-pandemic circumstances. Although, in Turkey, hospital access was officially permitted during the pandemic, the government-imposed curfew for individuals over the age of 65, who represent the majority of patients receiving denosumab, may have prevented some patients from attending follow-up visits. A recent study from Japan has also reported a significant increase in postponed denosumab treatment during the pandemic (29).

Patients with multiple comorbidities are commonly believed to have lower medication compliance. However, in our study, the level of comorbidity was similar between the groups, consistent with previous findings (30). Additionally, adherence to denosumab was not influenced by age, BMI, the age at menopause, or occupation. The most significant factors affecting treatment compliance were high parity and widowhood. This may be attributed to the increased social and economic burdens associated with multiple childbirths. Additionally, the loss of a spouse may lead to the absence of reminder mechanisms for medication intake and a declined motivation for managing one’s health. Although previous studies suggested that patients with prior osteoporosis treatment were more adherent than treatment-naive patients (31), we did not find a significant difference in adherence rates between these groups.

The strengths of the study include reliable data collection due to our center’s extensive archive, including a fracture liaison service (FLS). FLS allows electronical recording of denosumab injections administered by an FLS nurse at the outpatient clinic, which eliminates any doubt regarding whether the patient withdrew medication from the pharmacy. Additionally, analyzing multiple VFx as a separate entity adds strength to the study. However, certain limitations should be considered. First, this study was a small, retrospective, and single-center analysis, which inherently limits the generalizability of the findings and may introduce selection bias or unmeasured confounding. Although we attempted to minimize selection bias by systematically reviewing all patient records for eligibility, we might not have been able to comprehensively control for all potential confounding factors. Second, since the study population primarily consisted of postmenopausal osteoporosis patients, the results may not be generalizable to all osteoporosis patients. Third, we were not able to reliably establish the exact timing of fractures due to missed follow-up visits during the pandemic, which precluded us from performing a detailed time-to-event analysis. Fourth, we did not assess BMD as a measure of therapeutic effect. Finally, as the data largely reflect the early post-pandemic period, our findings may underestimate denosumab adherence rates compared with regular health care periods. However, it is unlikely that the risk of new fracture development following denosumab discontinuation was significantly over- or underestimated.

In conclusion, our results indicate that although fractures after denosumab discontinuation may be attributed to the rebound effect, some may occur due to the inherent nature of osteoporosis. Nonetheless, our findings do not imply that discontinuing denosumab without subsequent therapy is safe, and unscheduled treatment discontinuation must be prevented.
